# Prospective analysis of circulating metabolites and endometrial cancer risk

**DOI:** 10.1016/j.ygyno.2021.06.001

**Published:** 2021-08

**Authors:** Laure Dossus, Eirini Kouloura, Carine Biessy, Vivian Viallon, Alexandros P. Siskos, Niki Dimou, Sabina Rinaldi, Melissa A. Merritt, Naomi Allen, Renee Fortner, Rudolf Kaaks, Elisabete Weiderpass, Inger T. Gram, Joseph A. Rothwell, Lucie Lécuyer, Gianluca Severi, Matthias B. Schulze, Therese Haugdahl Nøst, Marta Crous-Bou, Maria-Jose Sánchez, Pilar Amiano, Sandra M. Colorado-Yohar, Aurelio Barricarte Gurrea, Julie A. Schmidt, Domenico Palli, Claudia Agnoli, Rosario Tumino, Carlotta Sacerdote, Amalia Mattiello, Roel Vermeulen, Alicia K. Heath, Sofia Christakoudi, Konstantinos K. Tsilidis, Ruth C. Travis, Marc J. Gunter, Hector C. Keun

**Affiliations:** a Nutrition and Metabolism Section, International Agency for Research on Cancer, Lyon, France; b Cancer Metabolism and Systems Toxicology Group, Division of Cancer, Department of Surgery and Cancer, Imperial College, London, UK; c European Food Safety Authority, Via Carlo Magno 1A, 43126 Parma, Italy; d Cancer Epidemiology Program, University of Hawaii Cancer Center, Honolulu, HI, USA; e Nuffield Department of Population Health, University of Oxford, Oxford, UK; f Division of Cancer Epidemiology, German Cancer Research Center (DKFZ), Heidelberg, Germany; g Office of the Director, International Agency for Research on Cancer, Lyon, France; h Department of Community Medicine, Faculty of Health Sciences, University of Tromsø, The Arctic University of Norway, Tromsø, Norway; i Centre for Research in Epidemiology and Population Health, CESP, Université Paris-Saclay, UVSQ, Inserm U1018, Villejuif, France; j Gustave Roussy, Villejuif, France; k Department of Statistics, Computer Science and Applications “G. Parenti” (DISIA), University of Florence, Italy; l Department of Molecular Epidemiology, German Institute of Human Nutrition Potsdam-Rehbruecke, Nuthetal, Germany; m Institute of Nutritional Science, University of Potsdam, Potsdam, Germany; n Unit of Nutrition and Cancer, Cancer Epidemiology Research Program, Catalan Institute of Oncology (ICO), Barcelona, Spain; o Nutrition and Cancer Group, Bellvitge Biomedical Research Institute (IDIBELL), Barcelona, Spain; p Department of Epidemiology, Harvard T.H. Chan School of Public Health, Boston,USA; q Escuela Andaluza de Salud Pública (EASP), Granada, Spain; r Instituto de Investigación Biosanitaria ibs.GRANADA, Granada, Spain; s Centro de Investigación Biomédica en Red de Epidemiología y Salud Pública (CIBERESP), Madrid, Spain; t Department of Preventive Medicine and Public Health, University of Granada, Granada, Spain; u Public Health Division of Gipuzkoa, BioDonostia Research Institute, Donostia-San Sebastian, Spain; v Department of Epidemiology, Murcia Regional Health Council, IMIB-Arrixaca, Murcia, Spain; w Research Group on Demography and Health, National Faculty of Public Health, University of Antioquia, Medellín, Colombia; x Navarra Public Health Institute, Pamplona, Spain; y Navarra Institute for Health Research (IdiSNA) Pamplona, Spain; z Cancer Epidemiology Unit, Nuffield Department of Population Health, University of Oxford, Oxford, UK; aa Institute for Cancer Research, Prevention and Clinical Network - ISPRO, Cancer Risk Factors and Life-Style Epidemiology Unit, Florence, Italy; ab Epidemiology and Prevention Unit, Fondazione IRCCS Istituto Nazionale dei Tumori di Milano, Milano, Italy; ac Cancer Registry and Histopathology Department, Provincial Health Authority (ASP) Ragusa, Italy; ad Unit of Cancer Epidemiology, Città della Salute e della Scienza University-Hospital and Center for Cancer Prevention (CPO), Turin, Italy; ae Dipartimento di Medicina Clinica e Chirurgia, Federico II University, Naples, Italy; af Institute for Risk Assessment Sciences, Utrecht University, Utrecht, the Netherlands; ag Department of Epidemiology and Biostatistics, School of Public Health, Imperial College London, London, UK; ah MRC Centre for Transplantation, King's College London, London, UK; ai Department of Hygiene and Epidemiology, University of Ioannina School of Medicine, Ioannina, Greece

**Keywords:** Metabolomics, Amino acids, Lipids, Endometrial cancer, Obesity, BMI, body mass index, C0, free carnitine, CI, confidence interval, CVs, coefficients of variation, EPIC, European Prospective Investigation into Cancer and Nutrition, IARC, International Agency for Research on Cancer, LC-MS/MS, liquid chromatography-tandem mass spectrometry, LLOQ, lower limit of quantification, LOD, limit of detection, MHT, menopausal hormone therapy, NIST, National Institute of Standards and Technology, OR, odds ratio, SD, standard deviation, SM, sphingomyelin, SRM, standard reference material, ULOQ, upper limit of quantification, WC, waist circumference

## Abstract

**Background:**

Endometrial cancer is strongly associated with obesity and dysregulation of metabolic factors such as estrogen and insulin signaling are causal risk factors for this malignancy. To identify additional novel metabolic pathways associated with endometrial cancer we performed metabolomic analyses on pre-diagnostic plasma samples from 853 case-control pairs from the European Prospective Investigation into Cancer and Nutrition (EPIC).

**Methods:**

A total of 129 metabolites (acylcarnitines, amino acids, biogenic amines, glycerophospholipids, hexoses, and sphingolipids) were measured by liquid chromatography-mass spectrometry. Conditional logistic regression estimated the associations of metabolites with endometrial cancer risk. An analysis focusing on clusters of metabolites using the bootstrap lasso method was also employed.

**Results:**

After adjustment for body mass index, sphingomyelin [SM] C18:0 was positively (OR_1SD_: 1.18, 95% CI: 1.05–1.33), and glycine, serine, and free carnitine (C0) were inversely (OR_1SD_: 0.89, 95% CI: 0.80–0.99; OR_1SD_: 0.89, 95% CI: 0.79–1.00 and OR_1SD_: 0.91, 95% CI: 0.81–1.00, respectively) associated with endometrial cancer risk. Serine, C0 and two sphingomyelins were selected by the lasso method in >90% of the bootstrap samples. The ratio of esterified to free carnitine (OR_1SD_: 1.14, 95% CI: 1.02–1.28) and that of short chain to free acylcarnitines (OR_1SD_: 1.12, 95% CI: 1.00–1.25) were positively associated with endometrial cancer risk. Further adjustment for C-peptide or other endometrial cancer risk factors only minimally altered the results.

**Conclusion:**

These findings suggest that variation in levels of glycine, serine, SM C18:0 and free carnitine may represent specific pathways linked to endometrial cancer development. If causal, these pathways may offer novel targets for endometrial cancer prevention.

## Highlights

• Endometrial cancer is the most common gynecological cancer in developed countries.
• Metabolomics may uncover novel pathways linked to endometrial cancer.
• 853 endometrial cancer case-control pairs from EPIC underwent metabolomic profiling.
• Specific amino acids, sphingolipids & carnitine were linked to endometrial cancer.
• If causal, these pathways may offer novel targets for endometrial cancer prevention.

## 1 Introduction

Endometrial cancer is the sixth most common cancer among women with more than 380,000 new cases diagnosed worldwide in 2018 [1]. Due to the obesity epidemic and declining rates of hysterectomy the incidence of endometrial cancer has been growing in the past decades and this trend is projected to continue in the coming decades [[2], [3], [4]]. Known modifiable and non-modifiable risk factors explain 45% to 70% of endometrial cancer cases depending on their prevalence [[5], [6], [7]]. A worldwide analysis conducted on the burden of cancer cases attributable to high body-mass-index estimated that 34% of endometrial cancer cases in 2012 could be attributed to high BMI but that this proportion varied from 17% in very low human development index (HDI) countries to 42% in high HDI countries [8]. Mechanistically, dysregulation of several metabolic pathways have been linked with endometrial cancer development including exposure to high estrogen levels, hyperinsulinemia, or elevated chronic inflammation [[9], [10], [11]]. However, these pathways may only partly account for the biological mechanisms involved in endometrial cancer development and detailed metabolic profiling and assessment of metabolic intermediates could provide important new insights into endometrial tumorigenesis with implications for risk assessment and novel preventative strategies.

Metabolomics is a powerful high-throughput approach to identify metabolites or metabolic signatures that are associated with disease development and could help identify novel biological mechanisms involved in pathogenesis [[12], [13], [14], [15], [16], [17]]. Currently, few epidemiologic studies have investigated the association between circulating metabolites of major biochemical classes and subsequent risk of endometrial cancer, and all were case-control studies of small sample size with blood samples collected after diagnosis [[18], [19], [20], [21], [22]]. To address these gaps in the literature, we performed a targeted metabolomic study in pre-diagnostic blood samples from 853 case-control pairs from the European Prospective Investigation into Cancer and Nutrition (EPIC).

## 2 Methods

### 2.1 Study population

EPIC is an ongoing multi-center cohort study including approximately 520,000 participants recruited in the early 1990's from ten European countries [23]. At recruitment, detailed information was collected on dietary, lifestyle, reproductive, medical and anthropometric data and a baseline blood sample was collected from most participants. All participants provided written informed consent to participate in the EPIC study. This study was approved by the ethics committee of the International Agency for Research on Cancer (IARC) and all centers.

Subjects were selected among participants who provided a blood sample and were cancer-free (other than non-melanoma skin cancer) at recruitment into the cohort. Women who reported having undergone hysterectomy were excluded. Incident cancer cases were identified either through record linkage with cancer registries or through active follow-up. Women diagnosed with first primary epithelial invasive endometrial cancer were selected as cases. Cancers were coded according to the Third Edition of the International Classification of Diseases for Oncology. Type I histologies included endometrioid adenocarcinoma, adenosquamous carcinoma, adenocarcinoma with squamous metaplasia, adenocarcinoma not otherwise specified, adenocarcinoma in adenomatous polyp, mucinous adenocarcinoma, mucin-producing adenocarcinoma (codes 8380, 8560, 8570, 8140, 8210, 8480, 8481). The inclusion of adenocarcinoma not otherwise specified in type I is justified because endometrioid adenocarninoma is the most common type of adenocarcinoma. Type II histologies included squamous cell carcinoma, clear cell adenocarcinoma, mixed cell adenocarcinoma, serous cystadenocarcinoma, papillary serous cystadenocarcinoma (codes 8070, 8310, 8323, 8441, 8460). A total of 761 cases were classified as type I and 42 cases were classified as type II. Fifty cases with other or unknown histologies were not classified into either type (codes 8000, 8010, 8020, 8260, 8950, 8980).

For each EPIC center, end dates of the study period were defined as the latest dates of complete follow-up for both cancer incidence and vital status (dates varied between centers, from June 2008 to December 2012). The median follow-up time of the eligible participants was 12.1 years (5th–95th percentiles: 2.0–17.8 years).

For each endometrial cancer case, one control was chosen at random among appropriate risk sets using incidence density sampling, as previously described [9]. A total of 853 cases and 853 controls were included in the analysis.

### 2.2 Laboratory measurements

Targeted metabolomic analyses of plasma samples were conducted in the laboratory of Cancer Metabolism and Systems Toxicology (Imperial College London), by liquid chromatography-tandem mass spectrometry (LC-MS/MS) platform using a 1290 Agilent UPLC coupled to a QTRAP 4000 SCIEX mass spectrometer. Metabolite profiling was carried out using the Absolute*IDQ*® p 180 Kit (Biocrates Life Sciences AG, Innsbruck, Austria) following the sample preparation protocol recommended by the manufacturer.

Samples were prepared in 96-well plates (23 well plates in total) and matched case-control sets were measured on the same plate. Laboratory personnel were blinded to case-control status of the samples. Along with the study samples, four replicates of a standardized plasma sample, standard reference material (SRM 1950), purchased from National Institute of Standards and Technology (NIST), were dispersed between study samples in each batch. The analytical performance was assessed based on the intra- and inter-batch coefficients of variation, calculated for all metabolites measured in the NIST samples (Supplementary Table 1**)**.

C-peptide was measured in the same laboratory at IARC, in two phases: 378 samples were previously measured in 2007 by an immunoradiometric assay by Immunotech (Marseille, France) with CVs < 11% and 1260 samples were measured in 2019 by an ELISA assay by Mercodia (Uppsala, Sweden) with CVs <7%.

### 2.3 Filtering of metabolites

Out of the 188 metabolites included in the kit, 31 were not detected in plasma and 8 had inter- or intra-batch CVs > 20% (Supplementary Table 1). Values lower than the lower limit of quantification (LLOQ), or higher than the upper limit of quantification (ULOQ), as well as lower than plate-specific limit of detection (LOD) (for compounds measured with a semi-quantitative method) were considered to be outside of the measurable range. Metabolites were excluded from the statistical analyses if more than 20% of observations were outside the measurable range (*n* = 20, Supplementary Table 1). A total of 129 metabolites were finally retained for statistical analyses. Of these metabolites, 108 had all values in the measurable range. For the remaining 21 metabolites, values outside the quantifiable range (all lower than LLOQ or LOD) were imputed with half the LLOQ or half the plate-specific LOD, respectively. For those 129 metabolites the median intra-batch CV was 7.0 (5 CVs > 15%) and the median inter-batch CV was 8.8 (11 CVs > 15%).

### 2.4 Statistical analyses

Characteristics of cases and controls were described using mean and standard deviation (SD) or frequencies. Log-transformed metabolite concentrations were used in all analyses. We used conditional logistic regression to estimate the risk of endometrial cancer per standard deviation (SD) increase in log metabolite concentration. We also investigated associations between endometrial cancer risk and specific metabolite ratios and sums (listed in Supplementary Table 2). Models were further adjusted for body mass index (BMI) or waist circumference (WC). None of the additional potential confounders that were evaluated (listed in Table 1) changed parameter estimates by more than 10%. For these variables, missing values were assigned the median (continuous variables) or mode (categorical variables) if they represented less than 5% of the population, or were otherwise classified in a “missing” category (breastfeeding, ever use of MHT). Additional adjustment for C-peptide concentrations (standardized by phase of the measurements) as a biomarker of hyperinsulinemia was also performed. Multiple testing was addressed by controlling for family-wise error rate at α = 0.05 by permutation-based stepdown minP adjustment of *P*-values (Perm-Pvalues), as this method better accounts for the dependence of the tests [24,25].

Heterogeneity was investigated by menopausal status, use of exogenous hormones and fasting status at blood collection, age at diagnosis, time between blood collection and diagnosis, histological subtypes, self-reported diabetes, WC and BMI, by introducing interaction terms in the models and using likelihood ratio tests. For WC and BMI, unconditional logistic regression adjusted for each matching factor was used. Sensitivity analyses were performed by restriction of analyses to hormone non-users, fasting participants, non-diabetic participants, and cases diagnosed after the first two years following blood collection.

**Table 1:** Baseline characteristics of endometrial cancer cases and matched controls – mean (SD) or N (%).

Variable	*N*	Cases (*N* = 853)	Controls (*N* = 853)
Age at blood collection^a^	1706	54.7 (7.5)	54.7 (7.5)
Age at diagnosis	853	63.0 (7.9)	–
Time between blood collection and diagnosis (years)	853	8.3 (4.5)	–
Fasting status^a^	1676		
0–3 h		374 (44.7%)	375 (44.7%)
3–6 h		153 (18.3%)	154 (18.4%)
>6 h		310 (37.0%)	310 (36.9%)
Age at menarche (years)	1674	12.8 (1.5)	13.1 (1.6)
Age at first full term pregnancy (years)^b^	1428	25.1 (4.2)	25.1 (4.1)
Number of full term pregnancies^b^	1626	1.9 (1.3)	2.1 (1.3)
Ever use of oral contraceptives (OC)	1680	339 (40.5%)	419 (49.8%)
Menopausal status at blood collection^a^	1706		
Premenopausal	428	214 (25.1%)	214 (25.1%)
Postmenopausal	1030	515 (60.4%)	515 (60.4%)
Perimenopausal	248	124 (14.5%)	124 (14.5%)
Age at menopause (years)^c^	787	50.9 (4.1)	49.6 (4.3)
Ever use of menopausal hormone therapy (MHT)^c^	1011	193 (38.1%)	190 (37.6%)
Use of OC/MHT at blood collection^a^	1664	164 (19.7%)	164 (19.7%)
Smoking status	1667		
Never		538 (64.8%)	511 (61.1%)
Former		178 (21.4%)	178 (21.3%)
Smoker		115 (13.8%)	147 (17.6%)
Cambridge physical activity index	1666		
Inactive		242 (29.2%)	209 (25.0%)
Moderately inactive		286 (34.5%)	313 (37.4%)
Moderately active		185 (22.3%)	203 (24.2%)
Active		116 (14.0%)	112 (13.4%)
Alcohol at recruitment (g/day)	1702		
Non-drinker		194 (22.8%)	191 (22.4%)
>0–3		285 (33.6%)	269 (31.6%)
>3–12		212 (25.0%)	223 (26.1%)
>12–24		158 (18.6%)	170 (19.9%)
Educational level	1624		
primary/no schooling		349 (43.2%)	376 (46.1%)
technical/professional/secondary		325 (40.2%)	292 (35.8%)
longer education		134 (16.6%)	148 (18.1%)
Height (cm)	1706	160.7 (6.8)	161.0 (7.0)
Weight (kg)	1706	71.4 (13.4)	66.5 (10.7)
Body Mass Index (kg/m^2^)	1706	27.7 (5.4)	25.7 (4.1)
BMI (WHO categories)	1706		
Underweight (<18.5 kg/m^2^)		3 (0.3%)	8 (0.9%)
Normoweight (18.5–24.9 kg/m^2^)		300 (35.2%)	413 (48.5%)
Overweight (25–29.9 kg/m^2^)		308 (36.1%)	316 (37.0%)
Obese (≥30 kg/m^2^)		242 (28.4%)	116 (13.6%)
Waist circumference (cm)	1570	85.3 (12.4)	81.3 (10.5)
Hip circumference (cm)	1570	105.6 (10.8)	101.6 (8.5)
Waist/Hip Ratio	1570	0.8 (0.1)	0.8 (0.1)
Prevalent diabetes	1462	34 (4.6%)	26 (3.6%)

a matching factor.

b Among parous women*.

c Among postmenopausal women.

The main analyses described above were complemented by an analysis focusing on clusters of metabolites, where Principal component analysis was used to derive one ``representative” for each cluster [26]. We used BMI-residuals of each cluster representative as predictors in an L1-norm penalized conditional logistic regression model that was applied on 100 bootstrap samples. For each bootstrap sample, obtained by randomly sampling 853 matched case-control pairs with replacement from our original sample, a lasso (L1-norm penalized conditional logistic regression model) was applied with the penalty parameter set to the largest value such that the 5-fold cross validated error was within one standard error of the minimum (BoLasso method) [27]. The proportion of bootstrap samples for which the lasso produces a non-null coefficient for each cluster representative is a measure of our level of confidence in the fact that this particular metabolite or group of metabolites is associated with endometrial cancer, after adjustment for BMI and other relevant metabolites.

All statistical tests were two-sided. Analyses were conducted using the SAS (version 9.4, Copyright © 2017, SAS Institute Inc.) and R (packages Epi, NPC, ClustOfVar and cLogitL1) softwares [[28], [29], [30]].

## 3 Results

Endometrial cancer cases were on average 63 years old at diagnosis and were diagnosed 8.3 years after blood collection (Table 1). Compared to controls, cases were on average slightly younger at menarche and older at menopause, were less often oral contraceptive users and slightly more often MHT users. Cases also had higher BMI, waist and hip circumferences than controls.

**Fig. 1:**
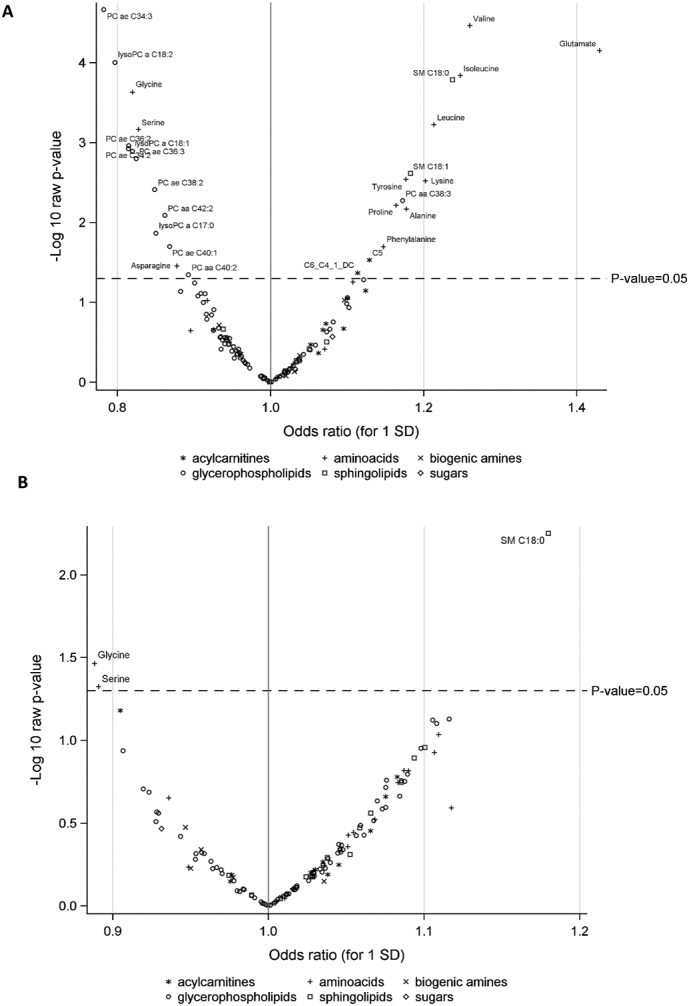
Odds ratios (ORs) and P-values for the associations between metabolites and risk of endometrial cancer in (A) unadjusted models (B) BMI-adjusted models. PC: phosphatidylcholine; SM: sphingomyelin. ORs are estimated per standard deviation (SD) increase in log-transformed metabolite concentrations, from logistic regression conditional on matching variables. Figs. A and B shows statistical significance based on *P*-values (significant metabolites above dotted line).

Twenty-eight metabolites were statistically significantly associated with endometrial cancer risk, including 12 amino acids, 12 glycerophospholipids, 2 acylcarnitines and 2 sphingolipids (Fig. 1A and supplementary Table 3a). After adjustment for BMI (Fig. 1B and supplementary Table 3b), two metabolites were statistically significantly associated with endometrial cancer risk: sphingomyelin [SM] C18:0 (OR_1SD_: 1.18, 95% CI: 1.05–1.33, *P*-value = 0.006, Perm-Pvalue = 0.38), and glycine (OR_1SD_: 0.89, 95% CI: 0.80–0.99, P-value =0.03, Perm-Pvalue = 0.87). Serine and free carnitine (C0) showed a borderline significant inverse association with endometrial cancer risk (OR_1SD_: 0.89, 95% CI: 0.79–1.00, P-value =0.05, Perm-Pvalue = 1.00 and OR_1SD_: 0.91, 95% CI: 0.81–1.00, P-value =0.07, Perm-Pvalue = 0.82, respectively). Similar results were observed after adjustment for WC (supplementary Table 3c) or C-peptide (supplementary Table 3d). None of these associations reached statistical significance after correction for multiple testing (Perm-Pvalues>0.38).

Cluster PCA identified 64 clusters (supplementary Table 4a), of which 3 (representing glycine, serine and SM C18:0 + SM C18:1) showed the strongest associations with endometrial cancer risk (supplementary Table 4b). Using the Bootstrap Lasso method, the following clusters were associated with endometrial cancer risk in more than 90% of the bootstrap samples (Glutamate+Taurine, Serine, C0, PCaa_C42:2, PC_aa_C42:5 + PCaa_C42:6, SM_C18:0 + SM_C18:1).

**Fig. 2:**
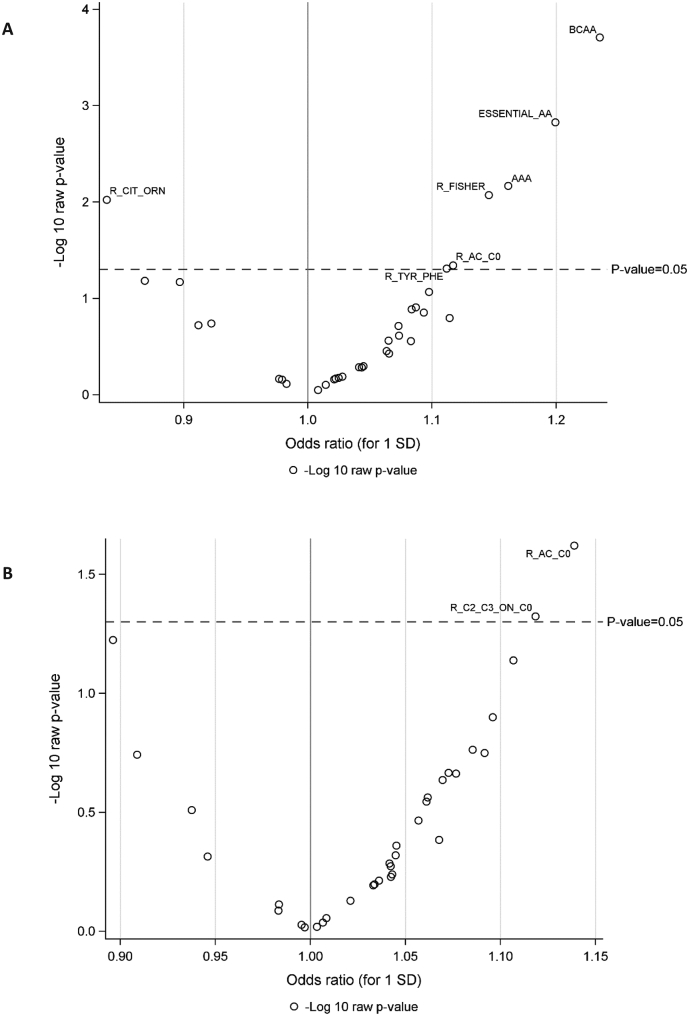
Odds ratios (ORs) and P-values for the associations between metabolite ratios and risk of endometrial cancer in (A) unadjusted models (B) BMI-adjusted models. ORs are estimated per standard deviation (SD) increase in log-transformed metabolite concentrations, from logistic regression conditional on matching variables. Figs. A and B shows statistical significance based on *P*-values (significant metabolites above dotted line).

Two metabolite ratios were positively associated with endometrial cancer risk after BMI adjustment (Fig. 2B and supplementary Table 3b): the ratio of esterified to free carnitine (OR_1SD_: 1.14, 95% CI: 1.02–1.28, *P*-value = 0.02, Perm-Pvalue = 0.31) and the ratio of short chain acylcarnitines to free carnitine (OR_1SD_: 1.12, 95% CI: 1.00–1.25, *P*-value = 0.05, Perm-Pvalue = 0.59).

Following restriction of the analyses to non-hormone users (*N* = 618 pairs), non-diabetics (*N* = 819 pairs) or to fasting samples (*N* = 310 pairs), similar BMI-adjusted estimates were observed although associations lost statistical significance because of reduced sample size in these sub-analyses. Associations with SM C18:0 were almost identical among non-hormone users and non-diabetics but were strongly attenuated and lost statistical significance when analyses were restricted to fasting samples (OR_1SD_: 1.10, 95% CI: 0.91–1.33, *P*-value = 0.32, Perm-Pvalue = 1.00). When cases diagnosed within 2 years after blood collection were excluded, slightly stronger associations were observed for SM C18:0 (OR_1SD_: 1.21, 95% CI: 1.07–1.37, *P*-value = 0.002, Perm-Pvalue = 0.20), glycine (OR_1SD_: 0.87, 95% CI: 0.77–0.97, P-value = 0.02, Perm-Pvalue = 0.68) and serine (OR_1SD_: 0.87, 95% CI: 0.77–0.98, P-value = 0.03, Perm-Pvalue = 0.97) and 2 additional amino-acids were positively associated with endometrial cancer risk, valine (OR_1SD_: 1.15, 95% CI: 1.01–1.30, P-value = 0.03, Perm-Pvalue = 0.77) and isoleucine (OR_1SD_: 1.14, 95% CI: 1.00–1.30, P-value = 0.04, Perm-Pvalue = 0.91).

No significant heterogeneity was observed for most metabolites when analyses were stratified by BMI (supplementary Table 3e and f), waist circumference, menopausal status, hormone use, histological subtype, age at diagnosis, lag time between blood collection and diagnosis or diabetes status (data not shown). Only serine showed a stronger association among cases diagnosed more than 8 years after blood donation (cases diagnosed ≤8 years: OR_1SD_: 1.03, 95% CI: 0.87–1.23, P-value = 0.70, Perm-Pvalue = 0.96; cases diagnosed >8 years OR_1SD_: 0.78, 95% CI: 0.67–0.92), P-value = 0.003, Perm-Pvalue = 0.82; P_heterogeneity_ = 0.03).

## 4 Discussion

In this large-scale prospective study of endometrial cancer, glycine, serine and free carnitine levels were inversely, and the sphingolipid SM C18:0 positively, associated with endometrial cancer risk even after control for BMI and other endometrial cancer risk factors. In addition, the ratio of esterified to free carnitine and the ratio of short chain acylcarnitines to free carnitine were both positively associated with endometrial cancer risk. However, none of these associations remained statistically significant following control for multiple comparisons.

Very few studies have evaluated metabolite profiles in relation to endometrial cancer risk and all were case-control studies of small sample size (number of endometrial cancer cases varying from 30 to 250) [[18], [19], [20], [21], [22]]. All studies but one [19] used different assays or methods to measure metabolites, potentially complicating the comparison between studies and with our results. Nevertheless, all studies observed specific metabolic alterations among cases compared to controls and three studies observed significant differences in amino acid levels although specific amino acids identified varied in each study [[20], [21], [22],^31^].

In this analysis we observed a potential inverse association between glycine and serine and endometrial cancer risk. Glycine and serine are non-essential amino acids which, in addition to their function in protein formation, play critical roles in metabolic regulation including one‑carbon metabolism [32]. Low plasma glycine levels have consistently been observed in individuals with obesity, insulin resistance and type 2 diabetes which are important risk factors for endometrial cancer [[33], [34], [35]]. It is therefore possible that glycine may be related to endometrial cancer through modification of insulin signaling. However, adjustment for C-peptide concentrations only slightly attenuated risk estimates, indicating a potential insulin-independent effect. Perturbations of glycine and serine metabolism are a common phenomenon in cancer development and tumours have an elevated demand for these amino acids [32]. In this study, similar estimates were observed for glycine and serine even after exclusion of cases that arose within 2-years after blood collection suggesting that the presence of sub-clinical disease was unlikely to be a major determinant of the observed relationship. Further work is now needed to explore whether glycine and serine play a direct causal role in endometrial cancer.

The potential positive association between SM C18:0 and endometrial cancer risk is in line with a recent report of a positive association of SM C18:0 with the endometrioid subtype of ovarian cancer in a prospective cohort [36]. Associations between sphingomyelins and diabetes and insulin resistance have also been observed and may partly explain their association with endometrial cancer [37,38]. Alterations of sphingolipid metabolism have been previously shown in endometrial cancer tissue compared to healthy endometrium [39] as well as in plasma of endometrial cancer patients compared to controls [19]. Interestingly, serine which showed an inverse relationship with endometrial cancer risk in our study forms part of the sphingolipid backbone. Sphingomyelins can be converted into ceramides that are involved in cell proliferation, migration and autophagy which may also explain their potential role in carcinogenesis [[40], [41], [42]].

We also observed a potential inverse association of free carnitine C0 with endometrial cancer risk and positive associations with the ratio of esterified to free carnitine and of short chain acylcarnitines to free carnitine. Carnitine plays an important role in the transport of long chain fatty acids into the mitochondrial matrix [43] and the ratio of short chain acylcarnitines to free carnitine is considered to be a measure of overall ß-oxidation activity. Moreover, the ratio of esterified to free carnitine is elevated in patients with type 2 diabetes and carnitine administration has been shown to improve insulin-mediated glucose disposal and storage in both diabetics and non-diabetic individuals [44]. The inverse association with C0 observed in our study may therefore reflect improved insulin sensitivity in women with elevated C0. Interestingly, a previous study conducted in the EPIC cohort reported a positive association between the acylcarnitine C2 and breast cancer risk [45]. These studies implicate a potential role for carnitine metabolism in obesity-related cancers which requires replication in other cohorts and mechanistic exploration.

Strengths of this investigation include the large number of incident endometrial cancer cases with pre-diagnostic specimens and extensive data on endometrial cancer risk factors. In addition, we were able to assess potential reverse causation by stratifying the analyses by time between blood collection and diagnosis. Our study also has limitations, particularly that the blood samples were collected from participants at one time point only. However, most of plasma metabolites analyzed have shown good reproducibility over time [46,47]. In particular, SM C18:0, glycine and C0 all had intra-class correlation coefficients >0.6 in samples collected 2 years apart. As our study has a mean follow-up of around 8 years, we cannot rule out some potential changes in some of the metabolites during follow-up that may have impacted the observed associations. Another limitation is that most of the measured compounds were quantified on a relative scale only because of lack of specific standards. Finally, none of the observed associations survived conservative adjustment for multiple testing, indicating that the potential associations, although biologically plausible, may be observed by chance due to the large number of tests conducted. Therefore, replication in future studies and in experimental models is now needed.

Compared to the untargeted approach, targeted metabolomics allows the measurements of quantified or semi-quantified identified compounds that facilitates comparability with other studies. The metabolites measured in the current study represent key biochemical pathways and have been previously associated with a number of chronic and metabolic diseases, including cardiovascular disease [48], metabolic syndrome [49], obesity [50,51], diabetes [52,53] and cancer [45,54,55]. Therefore, using this approach, we can target pathways that are potentially dysregulated in obesity and/or diabetes, two established risk factors for endometrial cancer as well as in cancer development. Further research with a fully untargeted platform would be an important next step to capture further novel pathways related to endometrial cancer development.

In conclusion, we demonstrate for the first time in a prospective study that alterations in concentrations of specific amino acids, sphingolipids and carnitine may be associated with endometrial cancer. If validated and shown to be causal, these findings may offer clues to novel etiologic pathways underlying endometrial cancer development.

### 4.1 Disclaimer

Where authors are identified as personnel of the International Agency for Research on Cancer / World Health Organization or the European Food Safety Authority, the authors alone are responsible for the views expressed in this article and they do not necessarily represent the decisions, policy or views of the International Agency for Research on Cancer / World Health Organization or the European Food Safety Authority.

## Funding

This work was supported by 10.13039/501100000289Cancer Research UK (CRUK) (grant number C19335/A21351, to MJG and HK).

The metabolomics infrastructure in the Division of Cancer, Imperial College London is supported by the 10.13039/100013216Imperial College Experimental Cancer Medicine Centre, the Imperial College Cancer Research UK Centre and the 10.13039/501100013342NIHR Imperial Biomedical Research Centre (APS & HK). The coordination of EPIC is financially supported by 10.13039/100008700International Agency for Research on Cancer (IARC) and also by the Department of Epidemiology and Biostatistics, School of Public Health, Imperial College London which has additional infrastructure support provided by the 10.13039/501100013342NIHR Imperial Biomedical Research Centre (BRC). The national cohorts are supported by: 10.13039/100008363Danish Cancer Society (Denmark); 10.13039/501100004099Ligue Contre le Cancer, Institut Gustave Roussy, 10.13039/501100008018Mutuelle Générale de l'Education Nationale, 10.13039/501100001677Institut National de la Santé et de la Recherche Médicale (INSERM) (France); German Cancer Aid, German Cancer Research Center (DKFZ), German Institute of Human Nutrition PotsdamRehbruecke (DIfE), Federal Ministry of Education and Research (BMBF) (Germany); Associazione Italiana per la Ricerca sul Cancro-AIRC-Italy, Compagnia di SanPaolo and National Research Council (Italy); Dutch Ministry of Public Health, Welfare and Sports (VWS), Netherlands Cancer Registry (NKR), LK Research Funds, Dutch Prevention Funds, Dutch ZON (Zorg Onderzoek Nederland), 10.13039/501100000321World Cancer Research Fund (WCRF), Statistics Netherlands (The Netherlands); Health Research Fund (FIS) - 10.13039/501100004587Instituto de Salud Carlos III (ISCIII), Regional Governments of Andalucía, Asturias, Basque Country, Murcia and Navarra, and the Catalan Institute of Oncology - ICO (Spain); 10.13039/501100002794Swedish Cancer Society, Swedish Research Council and County Councils of Skåne and Västerbotten (Sweden); 10.13039/501100000289Cancer Research UK (14136 to EPIC-Norfolk; C8221/A29017 to EPIC-Oxford), 10.13039/501100000265Medical Research Council (1000143 to EPIC-Norfolk; MR/M012190/1 to EPIC-Oxford). (United Kingdom).

## Declaration of Competing Interest

None.

## Appendix A Supplementary data

Supplementary Table 1 Completeness and exclusions of the different metabolites, with limits of detection (LOD) and limits of quantification (LOQ). Supplementary Table 1

Supplementary Table 2 List of common metabolite indicators proposed by Biocrates. Supplementary Table 2

Supplementary Table 3 Odds ratios (ORs) and P-values for the associations between metabolites and metabolite ratios and risk of endometrial cancer in (a) unadjusted models; (b) BMI-adjusted models; (c) models adjusted for wasit circumference; (d) models adjusted for BMI and C-peptide; (e) BMI-adjusted models and women with BMI less than 25; (f) BMI-adjusted models and women with BMI more than 25 Supplementary Table 3

Supplementary Table 4 a. Correlation coefficients between metabolites and PCA cluster representatives. b. Association between PCA clusters and endometrial cancer risk. Supplementary Table 4
